# The biodistribution of self-assembling protein nanoparticles shows they are promising vaccine platforms

**DOI:** 10.1186/1477-3155-11-36

**Published:** 2013-11-12

**Authors:** Yongkun Yang, Tobias Neef, Christian Mittelholzer, Elisa Garcia Garayoa, Peter Bläuenstein, Roger Schibli, Ueli Aebi, Peter Burkhard

**Affiliations:** 1 Department of Molecular and Cell Biology and Institute of Materials Science, University of Connecticut, 97 N. Eagleville Road, Storrs, CT 06250, USA; 2 Paul Scherrer Institute, Center for Radiopharmaceutical Science, Villigen, Switzerland; 3 M.E. Müller Institute for Structural Biology, Biozentrum, University of Basel, Basel, Switzerland

**Keywords:** Self-assembly, Nanoparticle, Bombesin, Drug-targeting, Vaccine, Biodistribution, Technetium

## Abstract

**Background:**

Because of the need to limit side-effects, nanoparticles are increasingly being studied as drug-carrying and targeting tools. We have previously reported on a scheme to produce protein-based self-assembling nanoparticles that can act as antigen display platforms. Here we attempted to use the same system for cancer-targeting, making use of a C-terminal bombesin peptide that has high affinity for a receptor known to be overexpressed in certain tumors, as well as an N-terminal polyhistidine tag that can be used for radiolabeling with technetium tricarbonyl.

**Results:**

In order to increase circulation time, we experimented with PEGylated and unPEGylated varities typo particle. We also tested the effect of incorporating different numbers of bombesins per nanoparticle. Biophysical characterization determined that all configurations assemble into regular particles with relatively monodisperse size distributions, having peaks of about 33 – 36 nm. The carbonyl method used for labeling produced approximately 80% labeled nanoparticles. *In vitro*, the nanoparticles showed high binding, both specific and non-specific, to PC-3 prostate cancer cells. *In vivo*, high uptake was observed for all nanoparticle types in the spleens of CD-1 nu/nu mice, decreasing significantly over the course of 24 hours. High uptake was also observed in the liver, while only low uptake was seen in both the pancreas and a tumor xenograft.

**Conclusions:**

The data suggest that the nanoparticles are non-specifically taken up by the reticuloendothelial system. Low uptake in the pancreas and tumor indicate that there is little or no specific targeting. PEGylation or increasing the amount of bombesins per nanoparticle did not significantly improve targeting. In particular, the uptake in the spleen, which is a primary organ of the immune system, highlights the potential of the nanoparticles as vaccine carriers. Also, the decrease in liver and spleen radioactivity with time implies that the nanoparticles are broken down and cleared. This is an important finding, as it shows that the nanoparticles can be safely used as a vaccine platform without the risk of prolonged side effects. Furthermore, it demonstrates that technetium carbonyl radiolabeling of our protein-based nanoparticles can be used to evaluate their pharmacokinetic properties *in vivo*.

## Background

There is a growing interest in nanoparticles and their various applications [1-4]. It is assumed that nanoparticles would bring about a better target to non-target ratio of drug uptake in drug delivery approaches. An interesting way to produce nanoparticles is the use of polypeptides, which are present in folded or unfolded form, depending on the nature and concentration of a salt in aqueous solution. The crucial point is that such peptides are selected, which stick together during the folding process in a well-defined amount and thus form a spherical nanoparticle [5]. We have used coiled-coil domains as building blocks to engineer self-assembling protein nanoparticles (SAPN) [6-8]. Such proteins can be produced with genetically engineered cells and can be extended at the N or C-terminus with freely selectable amino acid residues. If the peptide sequence of a known antigen is used, formation of the nanoparticle will result in the repetitive display of that antigen. We have used this concept to produce promising vaccine candidates for illnesses such as influenza, malaria, SARS, and HIV [9-12].

Here we tried to use the same system to develop nanoparticles for drug targeting and medical imaging. The N-terminus of the peptide chain usually comprises six histidines, a so-called His-tag, which is used to separate the engineered peptide on a Ni-NTA-column. The His-tag is not only a chelator for Ni on the column but has a high affinity for the Technetium tricarbonyl as well, and thus, it can be used for labeling with the Technetium carbonyl method [13-15]. At the C-terminus, in addition to linkers, we have introduced Asn-Gln-Trp-Ala-Val-Gly-His-Leu-Met, namely bombesin (BN) (residues 6–14). Bombesin was selected because it is known to have a high affinity to the GRP receptor, which is over-expressed in many tumors. Moreover, we have investigated the free bombesin peptide for many years [16,17]. Radiotracers consisting of biologically active compounds and radionuclides are powerful tools in three different areas of medical applications [18-21], namely diagnosis, therapy, and drug development. The latter gained increasing interest since the introduction of a radiolabeled drug analogue to study a limited number of patients or healthy volunteers gives insight into the properties and behavior of a drug in humans. Generally, positron emitters such as C-11 (at the place of C-12) or F-18 (substituting stable -H or -OH) are used for this purpose because the quantification of the organ uptake is much easier with positron emission tomography (PET) than with single-photon emission computed tomography (SPECT). However, the radiation burden with PET is rather high and considerable reduction could be achieved if a Technetium-99 m (^99m^Tc) labeled analogue was applied.

**Figure 1:**
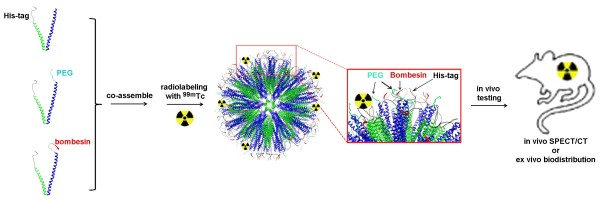
Schematic drawing of the co-assembly of the protein chains to multi-functional nanoparticles and the radio-labeling experiments.

The goal of this study was to assess the biodistribution of radionuclide-labeled self-assembling protein nanoparticles in a tumor mouse model. To this aim the protein chain of the nanoparticle was modified with the tumor targeting peptide bombesin and then co-assembled with the same protein chain without bombesin at different ratios. Nanoparticles are known to be trapped in the reticuloendothelial system (RES), mainly in the liver like the labeled colloids, which have been introduced in the nuclear medicine long ago. Since this elimination from the blood stream strongly hampers the uptake into other target tissues we also introduced poly(ethylene glycol) (PEG) coated particles, an approach that has previously successfully been used [22,23]. Radioactive technetium was then attached to the His-tag of the protein and the biodistribution was assessed in a mouse model (Figure 1). No increased accumulation of the radioactivity could be observed in tumor cells, even when the particles were PEGylated. However, the biodistribution strongly indicates that these nanoparticles are ideal vaccine candidates as they significantly accumulate in the spleen, which plays an important role in the immune system. These results support our previous findings [9-12], which demonstrate the nanoparticles’ ability to act as effective vaccines.

## Results

### SAPN design

**Figure 2:**
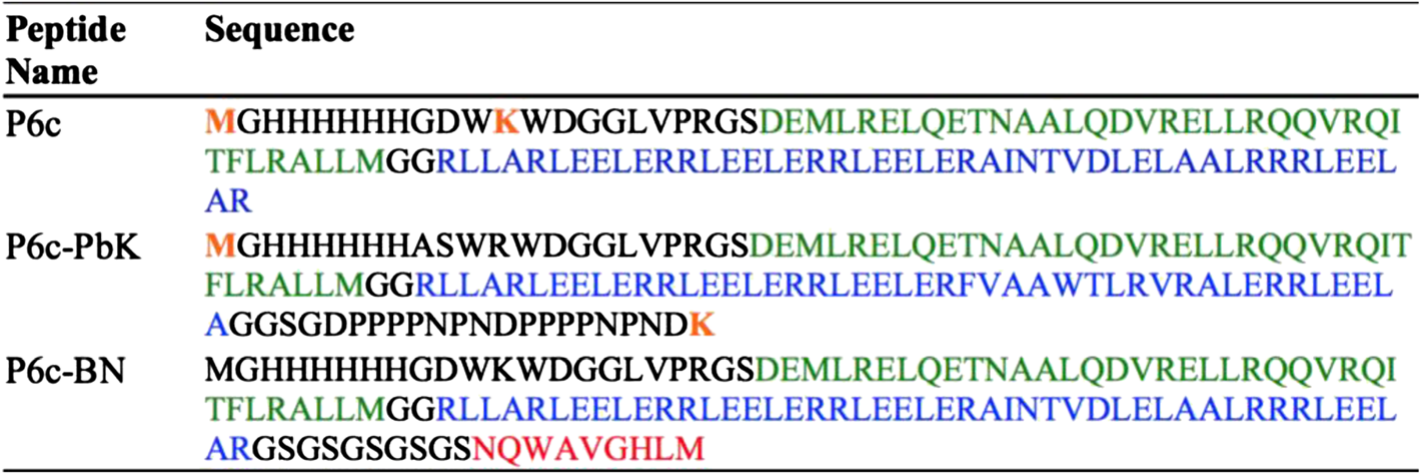
**Peptide sequences used.** Green = pentamer, Blue = trimer, Red = bombesin, Black = linkers and His-tag, Orange = amino groups used for PEGylation.

We wanted to test whether there was a difference in the biodistribution of the nanoparticles based on different density of the targeting peptide bombesin and different coupling modalities of PEG to the nanoparticles. Figure 2 shows the amino acid sequences of the three different peptides used in this study. The density of bombesin was varied by co-assembly of P6c (or P6c-PbK) with P6c-BN at different ratios (Table 1), while the PEG moiety was either attached to P6c or P6c-PbK. Both P6c and P6c-PbK peptides have two primary amine groups (α-NH_2_ from the methionine residue, and ϵ-NH_2_ from the lysine 12 in P6c and lysine 127 in P6c-PbK, respectively, all depicted in orange in Figure 2) in each chain. However, the lysine side chain in P6c is located between two bulky tryptophan residues and might therefore not be accessible enough for efficient coupling of the PEG moiety. Also, in P6c the two coupling sites are both located on the N-terminal end of the nanoparticle peptide chain. Therefore, in the P6c-PbK peptide the lysine coupling site was engineered to the C-terminus at the Therefore, in the P6c-PbK peptide the lysine coupling site was engineered to the C-terminus at the very end of a peptide surface modification (GGSGDPPPPNPNDPPPPNPND) that previously produced very nice nanoparticles [24], hence it was moved from a less exposed site on the N-terminus to a well-exposed site on the C-terminus. It was expected that in the P6c-PbK design the coupling efficiency is higher and that the PEG moiety is more surface exposed on the nanoparticles.

**Table 1:** Refolding ratios of the different peptides without BN and P6c-BN

**Refolding trial**	**P6c**	**P6c-PEG**	**P6c-PbK-PEG**	**P6c-BN**	**Name**
1	0	0	50	10	P6c-PbK-PEG/P6c-BN 50:10
2	0	0	40	20	P6c-PbK-PEG/P6c-BN 40:20
3	0	50	0	10	P6c-PEG/P6c-BN 50:10
4	50	0	0	10	P6c/P6c-BN 50:10

### PEGylation of P6c and P6c-PbK

**Figure 3:**
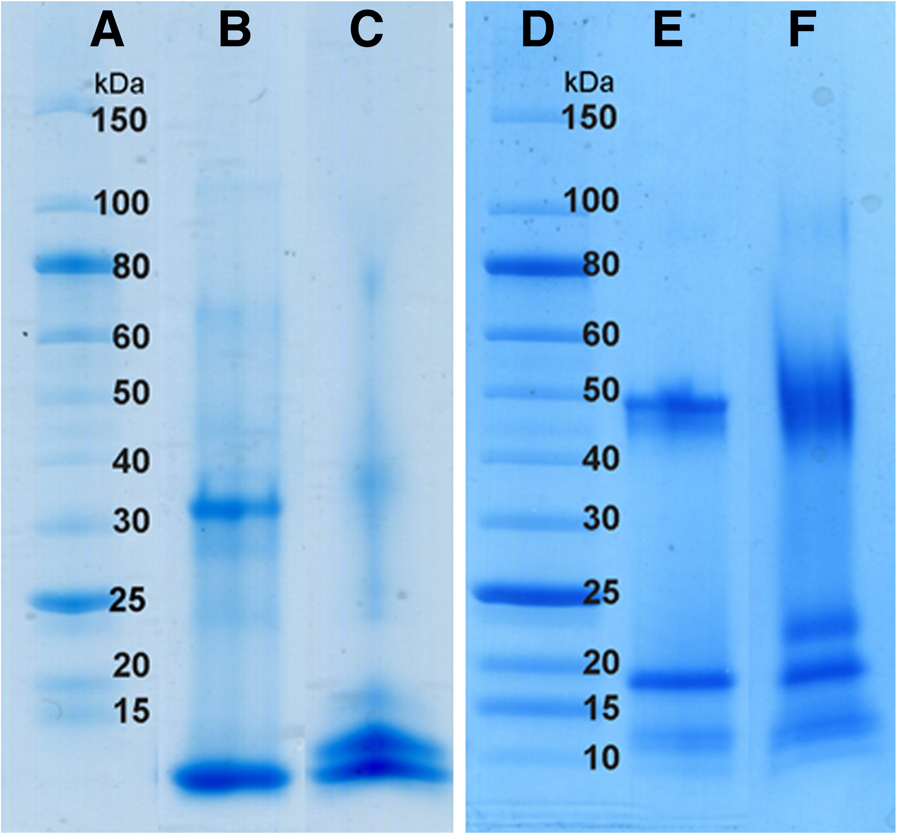
**SDS-PAGE analysis of the PEGylation products.** Left, the P6c peptide: **A)** Mw Marker **B)** uncoupled **C)** coupled with PEG; Right, the P6c-PbK peptide: **D)** Mw Marker **E)** uncoupled; **F)** coupled with PEG. For both constructs also the trimer band is visible on the gel.

In an attempt to increase the SAPN circulation time, we used PEGylated versions of the nanoparticle forming peptides. The P6c and P6c-PbK peptides were PEGylated using methoxyl PEG succinimidyl ester (mPEG-NHS) in denaturing conditions. The N-hydroxylsuccinimide (NHS) functional group reacts with the primary amine groups in the peptides. SDS-PAGE was used for analyzing the PEGylation products, as shown in Figure 3. The P6c PEGylation products have an extra band that ran slightly above the P6c monomer band (Figure 3, left panel, lane C), which indicated that some of the P6c peptide chains were PEGylated. The PEGylation ratio is about 30-50% by estimating the intensity of the two bands. As shown in Figure 3, right panel, the unPEGylated (lane E) and the P6c-PbK PEGylation (lane F) products showed similar results, except the extra band that ran above the P6c-PbK monomer band after PEGylation. This extra band is the trimeric form of the protein because it forms a stable coiled-coil even under the strongly denaturing conditions of the SDS-PAGE. The PEGylation ratio for the P6c-PbK peptide is also about 30-50 % by estimating the intensity of the two bands. The PEGylation reaction often produces a mixture of heterogeneous PEGylated compounds. It is difficult to determine the number and location of the PEG molecules from the SDS-PAGE analysis, and hence it is also difficult to detect a difference in the coupling efficiency between the two peptide chains.

### Particle size distribution

**Figure 4:**
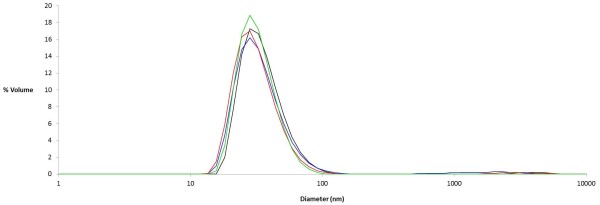
**Size distributions of different nanoparticle coassemblies as measured by dynamic light scattering.** Black: P6c/P6c-BN 50:10 (Peak = 36.4 nm). Blue: P6c-PEG/P6c-BN 50:10 (Peak = 34.8 nm). Red: P6c-Pbk-PEG/P6c-BN 50:10 (Peak = 32.8 nm). Green: P6c-Pbk-PEG/P6c-BN 40:20 (Peak = 33.1 nm).

**Figure 5:**
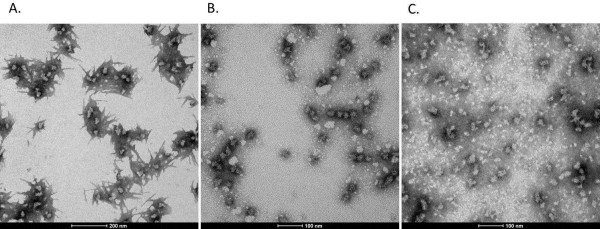
**Transmission electron microscopy of PEGylated samples. A)** P6c-Pbk-PEG/P6c-BN 50:10. **B)** P6c-Pbk-PEG/P6c-BN 40:20. **C)** P6c-PEG/P6c-BN 50:10.

When the particles were refolded using a stepwise procedure, dynamic light scattering showed a size distribution with a peak of about 35 nm (Figure 4). All 3 PEGylated samples had size distributions with surprisingly similar peak diameters. Transmission electron microscopy (Figure 5) confirmed that regular, spherical particles with sizes in this range formed. It is also apparent, however, that PEGylation led to staining artifacts in the image.

### Radiolabeling

The labeling procedure with the carbonyl method was not optimized and yielded up to 80 % of labeled particles. The impurities were not identified, however, the chromatogram obtained during purification showed both small and large impurities. The small ones were of similar size as pertechnetate, the large ones aggregates of a few nanoparticles. The labeled particles were stable for at least one day, corresponding to four half live times of ^99m^Tc.

### In vitro properties

The *in vitro* studies showed the same pattern in all experiments, namely a high total binding to the cells and an equally high non-specific binding. As a consequence the specific binding (difference of total and non-specific binding) reflected only the statistical error of the experimental data.

### In vivo properties

**Figure 6:**
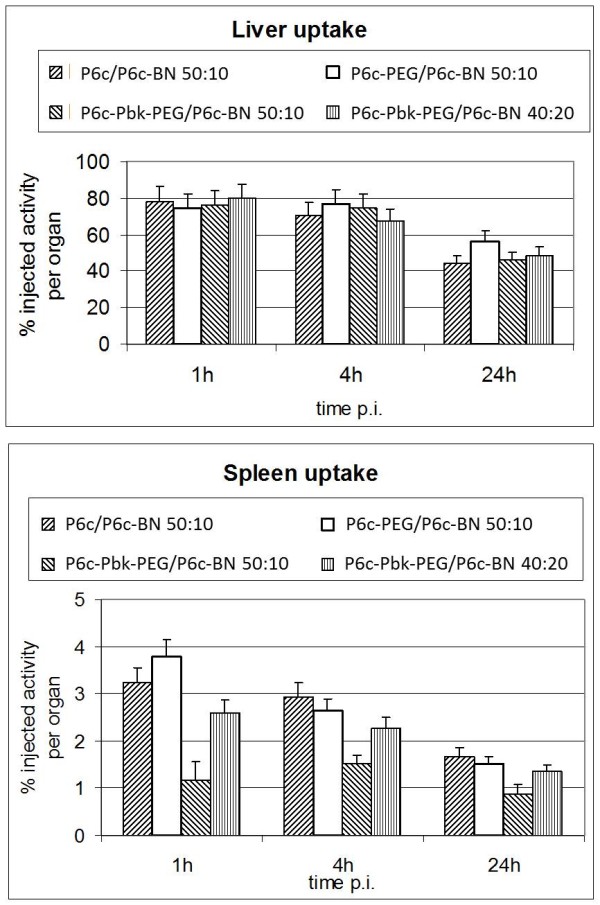
Uptake in the liver (top) and spleen (bottom) expressed as % of the injected activity per whole organ at different times after injection.

The biodistribution (Figure 6 and Table 2) showed high uptake in the liver and the spleen, which was quite similar at 1 h and 4 h post injection, while a clear decline was seen at 24 h. Roughly 80% (± 10%) of the injected activity was trapped in the liver (Figure 6). Since the weight of the liver showed inter-animal variation between 650 and 1200 mg, concentrations from 60% up to 140% injected dose per g tissue were obtained (Table 2). The uptake per whole spleen was only 1% of the injected dose in the case of P6c-PbK-PEG/P6c-BN 50:10; a slight increase at 4 h and decrease at 24 h post injection were seen (Figure 6). A diverging behavior concerns the time course of the % ID/g (Table 2). There the Tc-labeled P6c-PbK-PEG/P6c-BN 50:10 decreases strongly from 21% ID/g (at 1 h) to 9% ID/g (at 24 h). The reason is the variable weight of the spleen. In most experiments animals with light and heavy spleens were rather uniformly distributed among the type of particles and time points. Only in the case of P6c-PbK-PEG/P6c-BN 50:10 it happened that the ''1 h group'' had light spleens (average 77 g) while the ''24 h group'' had rather heavy spleens (average 121 g). The other three types of particles showed an uptake of 3% (± 1 %) at 1 h post injection. Specific bombesin mediated uptake would show elevated values of ^99m^Tc activity in the pancreas and the tumor. However, in agreement with the *in vitro* data this was not observed. The uptake in the stomach and colon was low, very close to that of the intestine at all time points. PEGylation did not appear to significantly affect the biodistribution.

**Table 2:** Biodistribution*

	**P6c/P6c-BN 50:10**			**P6c-PEG/P6c-BN 50:10**			**P6c-PbK-PEG/P6c-BN 50:10**			**P6c-PbK-PEG/P6c-BN 40:20**		
**Organ**	**1 h**	**4 h**	**24 h**	**1 h**	**4 h**	**24 h**	**1 h**	**4 h**	**24 h**	**1 h**	**4 h**	**24 h**
Blood	0.31(4)	0.25(8)	0.13(2)	0.5(1)	0.42(6)	0.17(2)	0.4(1)	0.4(1)	0.15(1)	0.33(2)	0.27(3)	0.14(1)
Heart	0.16(4)	0.13(2)	0.09(4)	0.20(4)	0.21(4)	0.12(4)	0.25(6)	0.21(4)	0.15(3)	0.21(3)	0.18(3)	0.15(5)
Lung	6(1)	2.8(7)	0.9(4)	4.8(3)	5(2)	1.0(2)	2.9(7)	1.8(3)	0.9(1)	6(3)	2.5(2)	1.01(2)
Spleen	37(10)	31(7)	19(4)	43(11)	27(6)	18(4)	21(3)	22(5)	9(1)	32(6)	33(8)	20(4)
Kidneys	2.5(2)	3.8(9)	4.5(1)	3.8(3)	7.4(9)	7(1)	3.0(8)	4.4(8)	3.5(3)	3.3(3)	4.3(1)	5.1(4)
Pancreas	0.4(6)	0.3(1)	0.05(5)	2(2)	0.23(1)	0.3(1)	0.17(5)	0.4(3)	0.3(2)	1(1)	1.1(9)	0.12(7)
Stomach	0.6(7)	0.26(2)	0.20(3)	0.8(1)	0.5(2)	0.27(7)	0.3(2)	0.3(2)	0.17(6)	0.8(4)	0.38(5)	0.24(7)
Intestines	0.23(4)	0.22(4)	0.21(6)	0.33(5)	0.26(2)	0.20(5)	0.4(2)	0.44(3)	0.25(5)	0.6(2)	0.34(6)	0.3(1)
Colon	0.25(9)	0.22(5)	0.3(1)	0.3(1)	0.30(7)	0.22(5)	0.25(6)	0.4(2)	0.15(4)	0.3(1)	0.26(3)	0.21(2)
Liver	78(7)	68(4)	40(11)	67(6)	66(5)	49(12)	103(34)	85(13)	38(3)	77(9)	73(17)	49(2)
Muscle	0.19(6)	0.12(4)	0.16(6)	0.6(4)	0.3(3)	0.2(1)	0.4(2)	0.3(2)	0.2(1)	0.9(5)	0.6(2)	0.09(1)
Bone	2.5(8)	1.6(5)	1.3(7)	3(1)	1.8(2)	1.6(4)	3(2)	1.9(5)	0.8(2)	2(1)	2.8(6)	1.5(3)
Tu (av)	0.12(2)	0.13(4)	0.13(6)	0.2(1)	0.2(1)	0.2(1)	0.16(6)	0.18(7)	0.09(4)	0.2(1)	0.13(2)	0.11(4)
Bile	6.6(7)	3(2)	9(7)	12(12)	8(7)	4(2)	8(3)	12(4)	15(10)	10(10)	12(8)	13(11)
Urine	19(10)	30(24)	21(2)	39(8)	57(9)	14(2)	23(20)	111(10)	17(2)	27(4)	30(5)	9(5)

*Data represent % injected dose per g tissue of four differently substituted SAPN at different time points after injection (the error of the last digit in parentheses).

## Discussion

Nanoscale assemblies represent a new and interesting approach to the development of vaccine technology. In particular, protein-based assemblies are an attractive choice for a vaccine platform because of their safety and flexibility of design [4]. The SAPN system we utilized in this work has the additional benefits of being easy to produce, biophysically well characterized [6-8], and able to elicit a response against even poorly immunogenic peptides [25]. Consequently, we have successfully used SAPNs as vaccines against a number of important diseases [9-12].

Genetically engineered protein nanoparticles and radiolabeling of their His-tag are tools to prepare particles of uniform size and to study the *in vivo* behavior. The nanoparticles showed a rather monodisperse size distribution that was not affected by coupling of PEG to the nanoparticles. The uptake pattern in liver and spleen (Figure 6) reflects a non-specific uptake in the RES, which is mainly depending on the size and maybe on the lipophilicity/hydrophilicity of the particle surface. Tröster et al. [26] have shown that lower spleen uptake correlates with higher lipophilicity. Surprisingly, we observed exactly the opposite: the more hydrophilic and hence less lipophilic P6c-PbK was less accumulated in the spleen than P6c (Figure 6). The peptide GGSGDPPPPNPNDPPPPNPNDK carries four charges and is quite hydrophilic. Furthermore, when incorporating a higher proportion of the hydrophobic bombesin into the nanoparticle, uptake in the spleen was increased.

While the liver uptake was around 80% of the injected dose 1 h after injection and showing virtually no variation, the uptake in the spleen was around 3% of the injected dose and considerably lower (1%) with the P6c-PbK-PEG/P6c-BN 50:10 particles. The observed uptake is quite similar to the distribution observed with ^99m^Tc labeled colloidal preparations, which is attributed to the properties of the RES. Depending on the nature of the colloids basically the size of the particles, increased uptake in either liver, spleen or bone marrow was seen. The uptake in the liver is quite high as would have been expected for protein-based materials. Unfortunately, the uptake could not be reduced by PEGylation of the nanoparticles but stays roughly the same for all the preparations used in this study.

The washout of the activity from liver and spleen can be interpreted as a degradation of the particles, which is well in agreement with the biological function of these organs. The total activity cleared via kidneys and urine could not be determined, however the activity concentration in the urine was generally around 10 to 20% of the injected dose per ml urine. Since the intact particles are roughly ten times larger than compounds, which would pass the glomerulum (cut off about 50 kDa or 4 nm diameter), this clearance must be due to broken down particles and short peptide chains. Another possibility would be simply a decomposition of the Tc-complex. However, if only the Tc-complex would be destroyed, the free Tc would be oxidized rapidly to pertechnetate which subsequently would lead to an increased uptake in the stomach. Such uptake was not observed. The *in vitro* tests showed a high binding to the cells, which increased with time. This time course was slightly different depending on the nature of the nanoparticles. However, no experiment showed any specific uptake, which could be attributed to the bombesin moieties on the particle surface.

## Conclusions

We have shown that self-assembling protein nanoparticles accumulate in the immune organ spleen and therefore have the potential to be very immunogenic, supporting our earlier research using SAPNs as vaccines. Our study also shows that the nanoparticles might be safely applied as the metabolism and elimination at least indicates that no long-term problems must be considered. In this respect we have also shown that radiolabeling of nanoparticles is a tool with high potential for performing pharmacokinetic studies of such nanoparticle vaccines. It should be noted that the originally planned application of the SAPNs presented here, namely drug targeting and imaging, does not appear feasible at this time, warranting further development.

## Methods

### Peptide design

The peptides P6c [6,8], P6c-PbK and P6c-BN consist of a pentameric coiled-coil domain derived from cartilage oligomeric matrix protein (COMP) [27], linked to a trimeric coiled-coil domain that was *de novo* designed [28,29]. The P6c peptide has a His-tag sequence (22 amino acids, black) at the N-terminus, followed by a pentameric domain (36 amino acids, green) and a trimeric domain (46 amino acids). The P6cPbK peptide was created based on the P6c peptide, but with a lysine residue at its C-terminus. In the peptide P6c-BN, the sequence was extended at its C-terminal end with a flexible linker (GSGSGSGSGS) and nine residues (NQWAVGHLM) from the peptide bombesin. The sequences of P6c, P6c-PbK and P6c-BN are shown in Figure 2.

### Transformation

Plasmids carrying the genes for P6c and P6c-BN were transformed into competent BL21(DE3) pLysS *E. Coli* cells (Novagen, Madison, WI) by adding 5 μL plasmid to 100 μL cells. After allowing the mixture to cool on ice for 30 minutes, the cells were treated with a heat shock at 42°C for 90 seconds. After cooling the cells on ice for 2 minutes, 800 μL SOC medium were added and the cultures grown at 37°C, under shaking at 300 RPM, for 45 minutes. 100 μL of the cultures were spread on LB-agar plates containing 100 μg/mL ampicillin and 30 μg/mL chloramphenicol and left overnight at 37°C.

### Peptide expression and purification

Peptides were expressed by inoculating 50 mL Luria broth containing 100 μg/mL ampicillin and 30 μg/mL chloramphenicol with a single, isolated colony from the transformation plate. The culture was grown overnight at 37°C. The following day, 30 mL were used to inoculate 3 L of Luria broth containing 100 μg/mL ampicillin and 30 μg/mL chloramphenicol. The cells were allowed to grow at 37°C, under shaking at 180 RPM, until the OD_600_ = ~0.5. Expression was induced by addition of isopropyl β-D-thiogalactopyranoside. The cells were grown for an additional 4 hours and harvested by centrifugation at 4,000 RPM for 15 minutes. Cell pellets were stored at -20 °C until purification.

Cells were resuspended in lysis buffer (9 M urea, 10 mM Tris pH 8.0, 100 mM NaH_2_PO_4_) and lysed by sonication. The lysates were clarified by centrifugation at 30,500xg for 45 minutes. Cleared lysate was then passed through a nickel affinity column (GE Healthcare, Waukesha, WI), which was washed first with lysis buffer then with high phosphate buffer (9 M urea, 10 mM Tris pH 8.0, 500 mM NaH_2_PO_4_, 20 mM imidazole). The column was then washed with 9 M urea, 20 mM citrate, 100 mM NaH_2_PO_4_, 20 mM imidazole, at three different pH values: 6.3, 5.9, and 4.3. Finally, the column was washed with lysis buffer containing increasing concentrations of imidazole. Purity of the collected fractions was determined by SDS PAGE.

The purified peptide was then dialyzed into imidazole-free buffer: 8 M urea, 20 mM Tris pH 7.5, 150 mM NaCl, 2 mM EDTA.

### PEGylation of P6c and P6c-PbK

The PEGylation agent mPEG-NHS (1 kDa) was purchased from Nanocs, Inc., New York, USA. The peptides were concentrated to 1–5 mg/ml in a buffer containing 50 mM sodium phosphate, pH7.5, 0.05% SDS. Then the mPEG-NHS powder was added to the concentrated peptide solution in 50 mM sodium phosphate, pH 7.5, 0.05% SDS and incubated with 1000 rpm shaking at room temperature. In the reaction mixture, the molar ratio of mPEG-NHS to peptide was about 100:1. After the PEGylation reaction, the excess of mPEG-NHS was removed using a dialysis membrane with MWCO of 6000–8000 kDa (Spectrum Laboratories, Inc., CA, USA). The product mixture was analyzed using SDS-PAGE. They were used without further purification.

### Refolding

For the un-PEGylated particles, the two peptides P6c and P6c-BN were first dialyzed into a denaturing buffer (8 M urea, 20 mM Hepes pH 7.5, 150 mM NaCl, 5% glycerol) then filtered with a 0.1 μm filter (Millipore, Billerica, MA). They were then mixed so that the ratio of moles P6c to moles P6c-BN was 50:10. The mixed peptide solution was then diluted with filtered denaturing buffer such that the final peptide concentration was approximately 0.1 mg/ml. Refolding was done by stepwise dialysis into buffers with 20 mM Hepes pH 7.5, 150 mM NaCl, 5% glycerol and successively lower urea concentration. The steps were: 6, 4, 2, 1, 0, 0 M urea. 0 M urea was used twice to ensure removal of any residual urea.

For the PEGylated samples the PEGylated products from the previous steps were directly used for co-assembling with unPEGylated P6c-BN without further purification. The PEGylated products were filtered and then denatured in the same denaturing buffer as is described above. They were then mixed to the appropriate molar ratios with unPEGylated P6c-BN (Table 1) and diluted to approximately 0.1 mg/ml. Refolding was performed by direct dialysis into 0 M urea buffer. The 0 M urea step was performed twice to remove residual urea.

### Dynamic light scattering

Dynamic light scattering experiments were carried out on a Zetasizer Nano S Instrument (Malvern, Worcestershire, UK), with a 633 nm He-Ne laser. All measurements were carried out at 25°C in 20 mM Hepes pH 7.5, 150 mM NaCl, 5% glycerol.

### Transmission electron microscopy

Samples were negatively stained with 1% uranyl acetate (SPI Supplies, Westchester, PA, USA) and observed with a FEI Tecnai T12 S/TEM at an accelerating voltage of 80 kV (FEI, Hillsboro, Oregon).

### Labeling with ^99m^Tc

Labeling with ^99m^Tc was done in two steps. First, 1 ml of [^99m^Tc]pertechnetate, obtained from a Mo/Tc-Generator (Ultratechnecow, Mallinckrodt), was added to a mixture of 4.5 mg sodium boranocarbonate, 2.9 mg borax and 9 mg sodium potassium tartrate and heated at around 95°C for 20 min. The obtained solution with [Tc(CO)_3_(H_2_O)_3_]^+^ was cooled to room temperature, neutralized with hydrochloric acid and buffered with phosphate buffer at pH 6–7. In a second step 10 – 50 μl of this solution (depending on the activity concentration of the generator eluate) was mixed with 0.1 ml of a suspension of 0.8 mg/ml of the respective SAPN and kept at 40–50°C for 1 h. After cooling, the final product was purified over a PD10 column since the labeling yield was only up to 80%. Two flow-through cells, one equipped with an UV detector, the other with a radioactivity detector, were used to analyze the eluate.

### Cell line for pharmacological tests

The human prostate adenocarcinoma cell line PC-3 was obtained from the European Collection of Cell Culture (CRL-1687, ECACC; Salisbury, England). The cells were maintained in DMEM GLUTAMAX-I supplemented with 10% FCS, 100 IU/mL penicillin G sodium, 100 μg/mL streptomycin sulfate and 0.25 μg/mL amphotericin B. The cells were incubated at 37°C in an atmosphere containing 5% CO_2_ and twice weekly subcultured after detaching with trypsin/EDTA (0.25%).

### Internalization studies

For internalization, PC-3 cells at confluence were placed in 6-well plates and left to attach overnight. Cells were incubated with the labeled analogues (4 kBq) in culture medium for 0.5, 1, 2, 4 and 24 h at 37°C (final volume 1 mL/well). Nonspecific binding was determined with 10 μM unlabeled BN (residues 1–14) or unlabeled particles. After the different incubation times, cells were washed twice with cold PBS to discard unbound peptide. Surface-bound activity was removed by a 5-min acid wash (50 mM glycin-HCl, 100 mM NaCl, pH 2.8), which was twice applied at room temperature. Afterwards, the plates were washed with cold PBS and the cells were lysed with 1 M NaOH twice. Surface-bound and internalized radioactivity was measured in the gamma counter. Additional experiments were carried out with internalization inhibitors, such as sucrose 0.4 mM, Phenylarsinoxid 0.01 mM, Methyl-β-cyclodextrin 10 mM, Genistin 0.1 mM, Chloroquine 0.1 mM, Cytocalsin B 0.05 mM.

### Binding test

PC-3 cells were placed at different concentrations (0.0625 – 2 million cells/well) in 12-well plates. They were incubated for 2 h at 37°C with 20 kBq ^99m^Tc labeled SAPN-BN (250 μl). Nonspecific binding was determined in presence of 1 μmol/l unlabeled bombesin (1–14). After incubation the supernatant was discarded and the cells washed twice with PBS. To detach the cells the wells were washed 2 times with 500 μl 1 N NaOH and the activity measured in a -counter.

### Biodistribution studies

All animal experiments were conducted in compliance with the Swiss animal protection laws and with the ethical principles and guidelines for scientific animal experimentation established by the Swiss Academy of Medical and Natural Sciences. Biodistribution studies were performed with 6- to 8-week-old female CD-1 nu/nu mice (20–25 g) purchased from Charles River Laboratories (Sulzfeld, Germany). For the induction of tumor xenografts, each mouse received subcutaneously 8x10^6^ PC-3 cells in 150 μL culture medium without supplements. The tumors were allowed to grow for at least three weeks. On the day of the experiment, the mice (3 per group) received the labeled nanoparticles intravenously. At 1, 4 and 24 h post injection (p.i.), the animals were euthanized and dissected. Blood, tumors and various healthy tissues and organs were collected and weighed. The amount of radioactivity in each tissue was determined by measuring the samples with the gamma counter and decay corrected (reference time = time of injection). Results are expressed as percentage of injected dose per gram of tissue (%ID/g).

## Abbreviations

SAPN: Self-assembling protein nanoparticle; BN: Bombesin; PEG: Poly (ethylene glycol); PET: Positron emission tomography; SPECT: Single-photon emission computed tomography; RES: Reticuloendothelial system; NHS: N-hydroxylsuccinimide; 99mTc: Technetium-99 m; COMP: Cartilage oligomeric matrix protein.

## Competing interests

PBu and UA have an interest in the company Alpha-O Peptides that has patents or patents pending on the technology.

## Authors’ contributions

YY and TN synthesized particles and performed biophysical characterization; CM provided construct plasmids; EGG and PBl performed radiolabeling, *in vitro*, and *in vivo* studies; RS, UA and PBu developed SAPN concept and gave direction for experiments. All authors contributed to the planning, writing, and reviewing of the manuscript. All authors read and approved the final manuscript.
